# Glatiramer Acetate Therapy Induces DNA Methylation Changes in Immune Cells of Multiple Sclerosis Patients: A Pilot Study

**DOI:** 10.3390/ijms27104615

**Published:** 2026-05-21

**Authors:** Ivan Kiselev, Olga Kulakova, Olga Baturina, Marsel Kabilov, Alexey Boyko, Olga Favorova

**Affiliations:** 1 Laboratory of Medical Genomics, Pirogov Russian National Research Medical University, 117997 Moscow, Russiaolga.favorova@gmail.com (O.F.); 2 Laboratory of Functional Genomics of Cardiovascular Diseases, Chazov National Medical Research Center of Cardiology, 121552 Moscow, Russia; 3 Genomics Core Facility, Institute of Chemical Biology and Fundamental Medicine, 630090 Novosibirsk, Russiakabilov@niboch.nsc.ru (M.K.)

**Keywords:** multiple sclerosis, disease-modifying therapy, glatiramer acetate, DNA methylation, epigenome-wide association study, peripheral blood mononuclear cells

## Abstract

Glatiramer acetate (GA) is a first-line disease-modifying therapy for multiple sclerosis (MS) with well-established moderate efficacy and high safety, yet its mechanisms of action remain incompletely understood. DNA methylation plays a significant role in MS development and is modulated by various environmental factors, including therapeutic drugs. In this pilot study, we report the first prospective analysis of genome-wide DNA methylation changes in peripheral blood mononuclear cells (PBMCs) from four female relapsing-remitting MS patients before GA initiation and after approximately four and eight months of therapy. We identified 365 loci that are characterized by differential methylation, distinguishing post-treatment time points from baseline, with significant enrichment in CpG islands, shores, and promoter regions. Two distinct temporal patterns emerged: (1) non-monotonic DNA methylation changes peaking at four months and associated with response to foreign antigenic stimuli, and monotonic changes progressively increasing by eight months and related to mTOR-associated pathways relevant to chronic inflammation and neurodegeneration. Integration of DNA methylation and transcriptomic data revealed significant methylation-expression correlations for eight genes, including HLA-*DMA*, *PDE4A*, and *SMOX*—genes with established roles in MS-associated antigen presentation, immunoregulation, and neuroinflammation. Cell composition of PBMCs remained stable throughout treatment. In general, GA therapy for MS appears to induce dynamic, locus-specific DNA methylation changes in PBMCs, with distinct temporal patterns suggesting a biphasic response of the immune system. However, given that none of the individual DMPs reached genome-wide significance, the results presented in this pilot study strongly require validation in larger independent cohorts. Nevertheless, we believe that our findings provide insights into the immunomodulatory effects of GA and lay the foundation for future hypothesis-driven studies to develop epigenetic biomarkers for therapeutic monitoring and generic GA product assessment.

## 1. Introduction

Multiple sclerosis (MS) is a chronic autoimmune, inflammatory, and degenerative disease of the CNS with polygenic inheritance, whose prevalence in Europe and North America is up to 250 persons per 100,000 [1]. MS primarily affects women of fertile age: the female-to-male ratio ranges from 2:1 to 3:1, while disease onset typically occurs between 15 and 45 years [1]. Although drugs for curative treatment of MS are still unavailable, disease-modifying therapy (DMT), which changes disease outcome through modifying autoimmune processes in MS patients, has existed since the early nineties when interferon-beta and glatiramer acetate (GA) were introduced for clinical practice [2]. To date, the FDA has approved numerous highly efficacious drugs, mostly based on monoclonal antibodies with different mechanisms of action [3]. Despite the modest efficacy of GA, high safety and systemic tolerance make it a good choice for the first-line therapy of MS with moderate activity [4]. The excellent long-term safety of GA makes it the primary option for patients with comorbidities, children, and the elderly, as well as for women during family planning, pregnancy, and lactation [5]. More than 20 years of use have made GA one of the most well-known DMTs with well-characterized possible adverse effects and proven treatment protocols.

GA is a synthetic polypeptide consisting of four randomly alternating amino-acid residues (L-glutamic acid, L-alanine, L-lysine, and L-tyrosine) in molar ratios equal to those in myelin basic protein [6]. Despite a long history of GA use, the mechanism of its action remains elusive. The central axis of its effect is believed to involve modulation of the interplay between T-cell populations and antigen-presenting cells. Presenting GA epitopes to T-cells may shift the immune system balance towards anti-inflammatory reactions, mainly through activating GA-reactive Th2- and Treg cells. They can further migrate into the CNS to suppress the activation of pro-inflammatory immune cells and the release of cytokines in situ [7].

Widespread use of the first approved GA product—Copaxone^®^, triggered development of multiple follow-on generic versions of GA, but often their similarity to Copaxone^®^ in efficacy, safety, and tolerability remains questionable [8]. In this situation, new methods suitable for the assessment of different GA generic products’ performance are sorely needed.

DNA methylation of CpG dinucleotides in the C5 position of a cytosine ring is one of the main epigenetic modifications, which can induce relatively stable alterations of gene expression in different cells and tissues [9]. Recent studies indicate that DNA methylation may play an important role in MS development and progression (for more details, see [10]). Although DNA methylation is considered a stable epigenetic modification, various environmental factors, including therapeutic drugs, can influence the epigenome over the course of a lifetime [11]. The effects of DMTs on DNA methylation profiles in MS patients have been demonstrated; however, the majority of the published works focus on therapy with Interferon-β. DNA methylation changes after GA treatment were analyzed in only two studies [12,13], which include independent samples of treated and untreated patients and provide very limited DNA methylation data.

The identification of differential DNA methylation patterns in MS patients undergoing GA therapy represents a promising avenue for elucidating novel mechanisms of drug action and for establishing objective criteria for assessing generic products’ performance. To this end, we present the first comparative analysis of genome-wide DNA methylation profiles in PBMCs from four female MS patients, assessed before the start of GA therapy and at the initial stages (~four and eight months of therapy). Furthermore, to investigate potential functional effects of differential DNA methylation on gene expression, we correlated DNA methylation changes with the expression levels of proximal genes, as measured by RNA sequencing.

## 2. Results

### 2.1. Estimated Immune Cell Proportions Do Not Change Significantly in MS Patients During Therapy

To investigate the potential role of DNA methylation in the response to GA, we conducted a genome-wide DNA methylation profiling in PBMCs of four relapsing-remitting female MS patients before the first administration of GA (time point “0 months”), as well as 4.2 ± 0.5 and 7.9 ± 0.5 months after therapy initiation (time points “4 months” and “8 months”, respectively).

**Figure 1:**
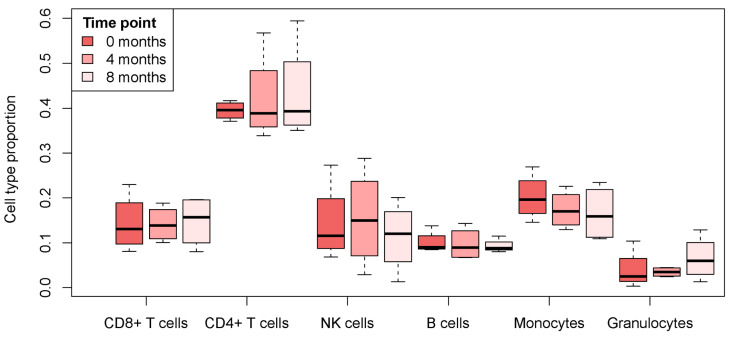
Boxplots of immune cell type proportions in PBMCs of MS patients before the first administration of GA (0 months), as well as after ~4 and ~8 months after therapy initiation.

To determine whether DNA methylation profiles were influenced by variations in the cellular composition of PBMCs, we estimated cell-type proportions (CD8+ and CD4+ T cells, NK and B cells, monocytes, and granulocytes) from the DNA methylation data. They did not differ significantly across the three time points (Figure 1 and Supplementary Table S1).

### 2.2. Glatiramer Acetate Therapy Induces Dynamic DNA Methylation Changes in MS Patients

**Figure 2:**
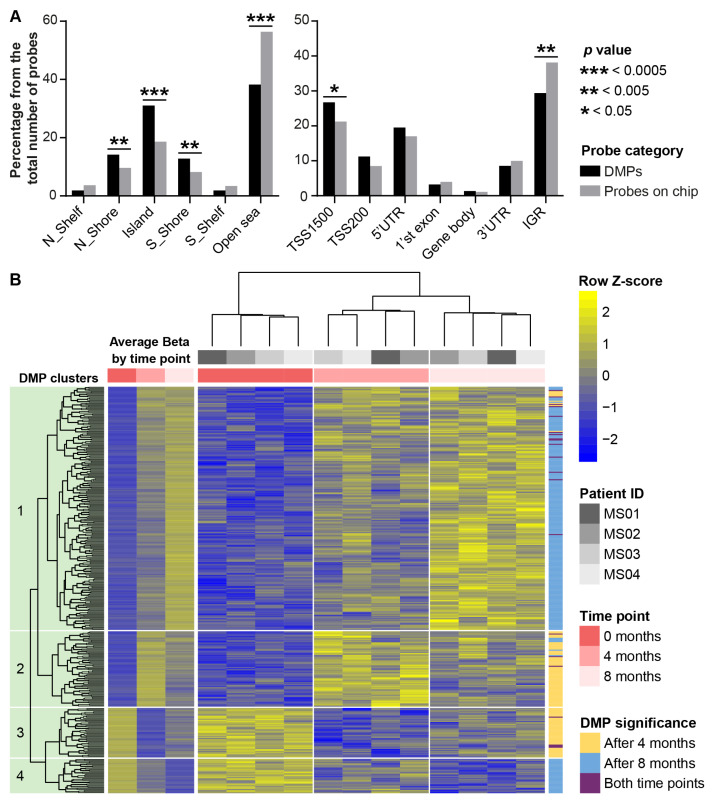
Visualization of the DMPs identified in PBMCs of MS patients on GA treatment. (**A**): Distribution of DMPs by CpG landmarks and gene structural elements. Each bar in the plots shows the ratio of the number of probes falling into the particular category to the total number of probes. TSS1500 and TSS200—regions within 1500–200 and 200–0 nucleotides upstream from the transcription start site (TSS), respectively; 5′UTR and 3′UTR—5′ and 3′ untranslated regions, respectively; IGR—intergenic regions. (**B**): Heatmap of z-scored methylation levels of identified DMPs. The color gradient from blue to yellow shows an increase in methylation level. Dendrograms showing the results of hierarchical clustering of samples and DMPs based on their similarity are shown above the heatmap and to the left of it, respectively.

Methylation data from 811,708 CpG-sites passed initial filtering. Genomic inflation factors (λ) calculated from the observed *p*-value distributions were 0.90 for the time point “four months” and 0.97 for the time point “eight months”, indicating a mild, conservative deflation consistent with the small sample size; the corresponding QQ plots are presented in Supplementary Figure S1. None of the differentially methylated positions (DMPs) crossed the genome-wide significance threshold *p* ≤ 5 × 10^−8^. 1317 DMPs passed the nominal significance threshold (*p* < 0.01, deltaBeta > 0.05) when time point “4 months” was compared to “0 months”, and 1951 DMPs when time point “8 months” was compared to “0 months”. To prioritize DMPs for subsequent analysis, we applied an additional filter of a limma posterior probability (B-statistic) > 0 (*p* ~ 0.0005 and less). This resulted in a final list of 365 DMPs (Figure 2 and Table S2). Of these, 110 DMPs were unique to the comparison “4 months vs. 0 months”, 234 were unique to the comparison “8 months vs. 0 months”, and 21 DMPs were common to both comparisons (see Table S2). No significant differentially methylated regions (DMRs) were identified using DMRcate and Bumphunter.

Genomic locations of the identified DMPs demonstrated significant enrichment with CpG-islands (*p* = 1.7 × 10^−7^) and their shores (*p* = 0.0037), as well as with regions proximal to gene promoters (TSS1500, *p* = 0.036) (Figure 2A). Conversely, open sea and intergenic genomic regions were significantly underrepresented (*p* = 3.5 × 10^−11^ and *p* = 0.0032, respectively).

Hierarchical clustering of DMPs (Figure 2B) demonstrates that samples group primarily by time point, reflecting treatment-associated shifts in methylation patterns. Four main clusters of DMPs based on similarities in their methylation dynamics were identified. The two largest clusters (clusters one and two in Figure 2B) comprise 288 DMPs (78.9% of the total), which were exhibiting hypermethylation during GA therapy. Two distinct temporal patterns of methylation changes (regardless of their direction) were observed: (1) non-monotonic therapy-associated changes (clusters two and three), which peaked at 4 months of therapy and subsequently regressed by 8 months, still not reaching baseline methylation levels (0 months) and (2) monotonic hyper- or hypomethylation (clusters one and four), whose magnitude increased from 4 to 8 months. These patterns are particularly evident when examining the average deltaBeta per time point (see Figure 2B).

### 2.3. DMP-Containing Genes Are Associated with Response to Antigenic Stimuli and Chronic Diseases

Of the 365 identified DMPs, 258 (70.7%) were located within the regions of known genes. This represents a statistically significant enrichment (*p* = 0.0005) compared to the total proportion of Infinium MethylationEPIC BeadChip probes located in gene regions (61.9%). Only two genes, *C5orf38* and *Y_RNA*, contained more than one DMP, with two DMPs identified in each case.

**Figure 3:**
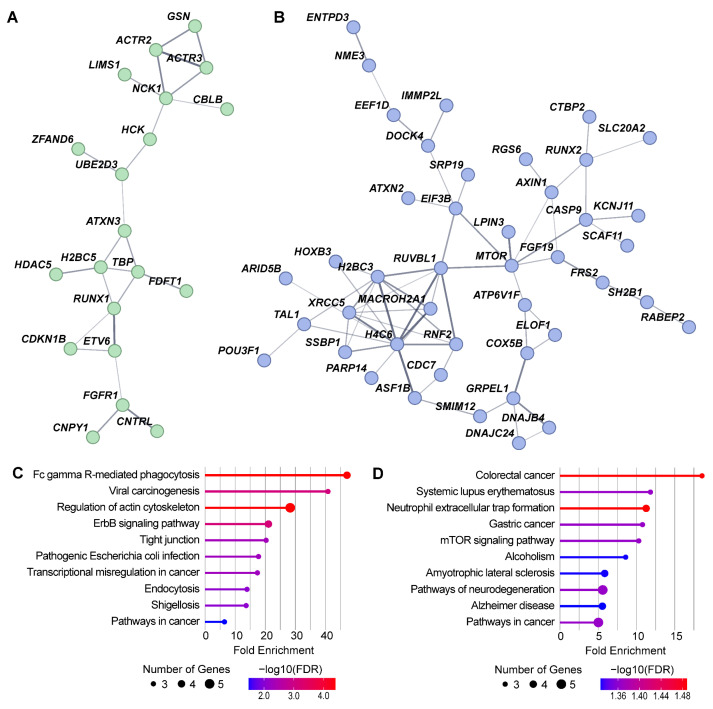
Functional annotation of DMP-containing genes with non-monotonic and monotonic temporal patterns of methylation changes during MS treatment with GA. (**A**) Largest connected component (LCC) of the STRING interaction network for genes with non-monotonic methylation changes. (**B**) LCC of the STRING interaction network for genes with monotonic changes. (**C**) KEGG pathways significantly enriched (p_adj_ < 0.05) in the LCC shown in (**A**). (**D**) KEGG pathways significantly enriched in the LCC shown in (**B**). In (**A**,**B**), edge line thickness indicates the strength of data support. In (**C**,**D**), line color indicates fold enrichment values, and circle size indicates the number of genes.

STRING network analysis of all DMP-containing genes revealed that 123 of these genes interact with each other (Figure S2), which is significantly more than expected by chance (protein–protein interaction (PPI) enrichment *p* = 0.031). To investigate the functional associations of DMP-containing genes exhibiting distinct temporal patterns of methylation changes (see Figure 3B), we performed separate STRING network and KEGG pathway enrichment analyses: genes from clusters two and three (non-monotonic changes) and genes from clusters one and four (monotonic changes) were analyzed independently (Figure 3 and Table S3).

The resultant network of DMP-containing genes exhibiting non-monotonic methylation changes contained the largest connected component (LCC) of 20 genes (Figure 3A). Members of this LCC were significantly enriched in KEGG pathways associated with phagocytosis, cytoskeleton regulation, and response to viral and bacterial infections (Figure 3C), indicating leukocyte activation in response to foreign antigenic stimuli. The LCC of genes with monotonic methylation changes comprised 43 genes (Figure 3B), which were significantly enriched by KEGG pathways related to mTOR-dependent signaling and associated with various chronic diseases accompanied by inflammatory, autoimmune, and neurodegenerative processes (Figure 3C).

### 2.4. Methylation Level of Several DMP-Containing Genes Correlates with Their Expression

**Figure 4:**
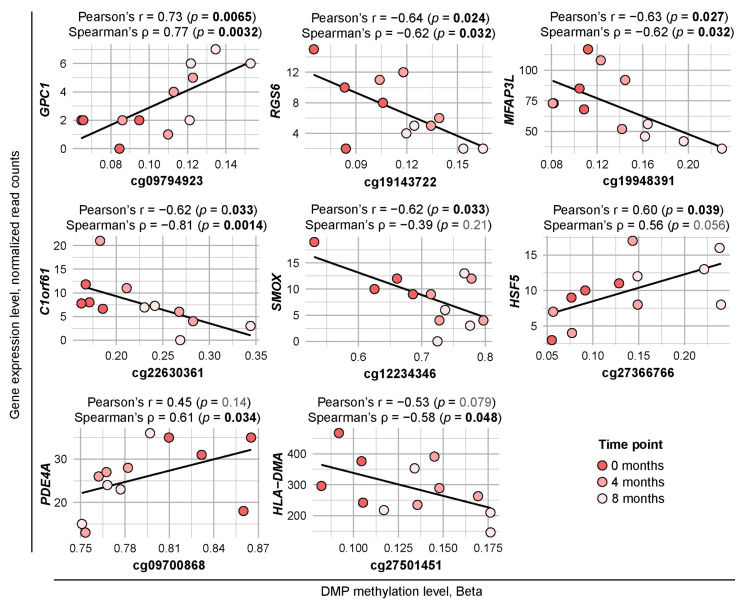
Significant correlations between DMP methylation levels and expression levels of DMP-containing genes. The black regression line is shown on each plot. Pearson’s and Spearman’s correlation coefficients and *p*-values calculated for each of DMP-gene pairs are presented above corresponding dot plots; significant *p*-values are marked in bold.

To investigate the potential correlation between methylation levels of DMPs and the expression of their corresponding genes, we performed transcriptome analysis on PBMCs from the same cohort of MS patients undergoing GA therapy. We identified eight genes with significant correlations between DMP methylation and expression levels (Figure 4, Table S4). The expression of five genes—*RGS6*, *MFAP3L*, *C1orf61*, *SMOX*, and *HLA*-*DMA*—decreased over the time course, which was associated with an increase in methylation of their DMPs. Conversely, the expression and methylation levels of *GPC1* and *HSF5* increased concurrently over time, while both the expression and methylation levels of *PDE4A* decreased. All eight DMPs were located within or in close proximity to gene promoter regions. Furthermore, all DMPs, with the exception of cg09700868 (*PDE4A*), were associated with known CpG islands.

## 3. Discussion

In this pilot hypothesis-generating study, we present the first prospective epigenome-wide analysis of DNA methylation changes in PBMCs from female MS patients undergoing GA therapy. By analyzing samples collected before treatment initiation and after approximately four months of therapy, we prioritized 365 DMPs that distinguish post-treatment time points from baseline. Our longitudinal design, incorporating two follow-up time points, enabled us to capture both transient and sustained epigenetic modifications, providing novel insights into the dynamics of GA-induced DNA methylation changes.

While the identified DMPs did not achieve genome-wide significance after multiple testing correction, the global methylation profiles clearly separated samples by treatment duration, reflecting consistent and coordinated GA-induced epigenetic changes across multiple loci. The failure to identify differentially methylated regions (DMRs) may reflect the relatively short follow-up period, as DMRs typically represent more stable epigenetic modifications that accumulate over longer timeframes. Indeed, a previous study examining GA-treated patients with a mean treatment duration of 56 months identified 26 DMRs in their T cells compared to untreated MS patients [13], supporting the suggestion that prolonged therapy is required for regional methylation changes to consolidate.

The predominance of DNA hypermethylation (78.9% of DMPs) contrasts with a previous report describing global DNA hypomethylation in PBMCs from GA-treated patients, as assessed by an ELISA-like approach [12]. This discrepancy likely reflects fundamental methodological differences: while global methylation assays provide a composite measure across all genomic contexts, our approach reveals region-specific GA-induced methylation changes in regulatory genomic compartments.

The observed methylation differences were not attributable to variations in PBMC composition, as estimated immune cell proportions remained stable throughout the eight-month treatment period. Previous studies on immune cell profiling in MS patients undergoing GA therapy have reported significant but not substantial changes in monocyte and B cell counts in peripheral blood, indicating that GA primarily modulates immune cell phenotype rather than overall composition [14,15,16]. These data support the assumption that the identified DMPs mostly reflect genuine therapy-induced epigenetic remodeling accompanying these functional changes. Moreover, the identified DMPs were significantly enriched in CpG islands and their shores, as well as in promoter-proximal genomic regions. This enrichment pattern aligns with the established role of promoter methylation in regulating gene expression and suggests that the observed epigenetic changes may influence immune cell activity.

Temporal clustering of DMPs revealed two distinct patterns of methylation dynamics: non-monotonic changes (hyper- or hypomethylation) that peaked at four months and partially regressed by eight months, and monotonic changes in DNA methylation that progressively increased in magnitude from four to eight months. Functional annotation of genes exhibiting distinct temporal patterns revealed that genes with non-monotonic methylation changes were enriched in pathways related to phagocytosis, cytoskeletal regulation, and immune responses to infections, implicating processes involved in initial leukocyte activation and antigen presentation. In contrast, genes with monotonic changes were predominantly associated with mTOR-dependent signaling cascades and pathways, which are known to be involved in various chronic inflammatory, autoimmune, and neurodegenerative diseases, including MS [17,18]. This biphasic pattern suggests that response to GA therapy may develop in two stages: an initial phase characterized by transient adaptations, representing acute responses to a new antigenic stimulus, followed by a sustained phase of progressive methylation changes directly associated with GA therapeutic effect. The identification of 21 DMPs common to both four- and eight-month comparisons (see Table S2) suggests a core set of differentially methylated regions that may be particularly sensitive to GA exposure and potentially contribute to early and sustained therapeutic effects.

Integration of epigenomic and transcriptomic data revealed significant correlations between DNA methylation and expression for eight genes, of which HLA-*DMA*, *PDE4A*, and *SMOX* contain core DMPs common to both comparisons (see Table S2). HLA-*DMA* merits special attention given its critical role in MHC class II antigen presentation: DM facilitates peptide loading by catalyzing the release of the CLIP molecule from the peptide-binding groove, thereby influencing the repertoire of antigens presented to CD4+ T cells [19]. The observed inverse correlation between HLA-*DMA* methylation and expression suggests that GA-induced hypermethylation may modulate autoantigen presentation capacity in MS [20], potentially contributing to the immunomodulatory effects of the drug. *PDE4A* encodes a phosphodiesterase involved in the degradation of cAMP, a key signaling molecule that regulates inflammation, immune responses, and cellular function. It represents another intriguing target for further research in the context of GA-based MS treatment, given the established immunomodulatory effects of PDE4 inhibitors [21]. Two such molecules, Rolipram and Ibudilast, were even considered promising candidate drugs for MS, with the latter successfully completing Phase II clinical trials in 2018 [22,23]. Finally, *SMOX* encodes a key enzyme in polyamine metabolism, and its elevated expression in activated macrophages contributes to pro-inflammatory phenotypes. SMOX inhibition exerts protective effects in experimental autoimmune encephalomyelitis, the primary mouse model of MS [24], suggesting that GA-induced downregulation of *SMOX* may influence redox balance in immune cells and thus represent one of the potential axes of GA action in MS.

Interestingly, for three of the eight DMP-containing genes presented in Figure 4—*PDE4A*, *GPC1*, and *HCF5*—we observed a direct correlation between promoter hypermethylation and increased gene expression. While known to be canonically associated with transcriptional silencing, emerging evidence from the 2020s reveals that increased promoter DNA methylation can also correlate with gene activation through several context-dependent mechanisms. Methylation may directly evict transcriptional repressors from bivalent chromatin domains or physically block the binding of methylation-sensitive repressor proteins, effectively relieving gene inhibition [25]. Methylation of a canonical gene promoter may force the use of alternative, more active promoters [26]. Finally, methylation-sensitive chromatin organizers such as CTCF may be blocked from forming repressive loops, allowing distal enhancers to activate the gene [27]. The specific mechanisms possibly responsible for the phenomena observed in our study should be investigated further.

Several limitations of this study should be acknowledged. The small sample size, while inherent to pilot investigations, limits statistical power and generalizability. Consequently, the 365 described DMPs should not be interpreted as definitive, validated treatment-induced changes. Instead, they represent a prioritized set of loci that exhibit consistent methylation differences in this longitudinal cohort and are suitable for generating biological hypotheses to be tested in larger, independent studies. The exclusive inclusion of female patients, although reflecting the higher prevalence of MS in women, precludes assessment of sex-specific epigenetic responses. It is known that seasonal variation, MS activity fluctuation, and biological aging can influence DNA methylation profiles [28,29,30], potentially biasing the obtained data. All these issues could be addressed if the study included a matched group of untreated MS patients. However, MS therapy now begins almost immediately after diagnosis, making the formation of such a group for a prolonged prospective observation nearly impossible. Nevertheless, patient ages (23 to 51 years) and the months of first blood sampling (March–October) in our cohort were desynchronized, making it unlikely that these factors could have biased the data. Moreover, all patients were in clinical remission at the start of therapy and did not experience clinical relapses during the observation period. Lastly, while estimated immune cell proportions did not change significantly over time in the studied patients, we cannot rule out residual confounding due to subtle shifts in cellular composition. Future studies on separate cell populations are needed to investigate the origin of the observed changes.

## 4. Materials and Methods

### 4.1. Participants and Study Workflow

Four female relapsing-remitting MS patients in clinical remission, aged 23 to 51 years, were enrolled in this study; none of them received any immunomodulatory drugs before the start of therapy. MS was diagnosed according to the McDonald Criteria (2017 revisions) [31]. Pregnant or lactating women, patients under 18 or over 59 years of age, and patients with mental illnesses or serious somatic diseases, whether infectious or non-infectious in nature, were not included in the study. All participants in the study were self-reported Russians and lived in the Moscow region. Main characteristics of the study participants are shown in Table 1.

**Figure 5:**
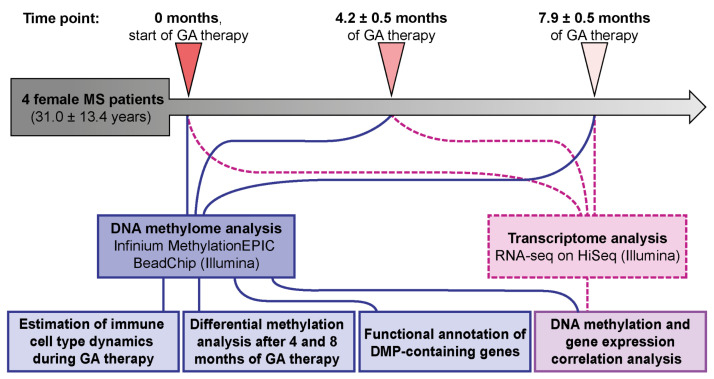
The workflow of the study.

The experimental workflow is outlined in Figure 5. Briefly, following enrollment in the study, participants provided a baseline blood sample in 9 mL EDTA tubes (Vacuette^®^, Greiner Bio-One, Kremsmünster, Austria) and immediately initiated GA therapy (Copaxone^®^, Teva Pharmaceutical Industries Ltd., Petah Tikva, Israel, 20 mg subcutaneously daily). Bedtime was recommended as optimal for GA administration to allow any injection-related side effects to subside overnight. Follow-up blood samples were collected prospectively at 4.2 ± 0.5 and 7.9 ± 0.5 months post-therapy initiation in the morning, approximately 8–12 h after the last GA injection. PBMCs were isolated within 2–3 h after blood collection by centrifugation on a Histopaque^®^-1077 Hybri-Max™ gradient (Sigma-Aldrich, St. Louis, MO, USA) and used for epigenome-wide DNA methylation and transcriptome analyses.

### 4.2. Epigenome-Wide DNA Methylation Analysis

Genomic DNA was isolated from PBMCs using the QIAamp DNA Mini Kit (Qiagen, Hilden, Germany); its purity and quantity were assessed using the NanoDrop 2000 spectrophotometer (Thermo Fisher Scientific, Waltham, MA, USA). Bisulfite conversion was performed using the EZ DNA Methylation-Gold Kit (Zymo Research, Irvine, CA, USA). DNA methylation levels were analyzed on the Infinium MethylationEPIC BeadChip using the iScan array scanner (Illumina, San Diego, CA, USA).

Raw methylation data processing was performed using the minfi R package v. 1.40.0. Quality control was assessed with the qcReport and plotQC functions of the minfi package; functional normalization was performed using the SWAN (Subset-quantile Within Array Normalization) method via the preprocessSWAN function of the same package. Probes with a detection *p*-value above 0.01, multi-hit probes, probes containing single nucleotide polymorphisms, and probes located on sex chromosomes (X and Y) were excluded from the subsequent analysis to avoid potential confounding effects. Cell type composition for each sample was estimated from the raw data using the estimateCellCounts function with an appropriate reference dataset. Statistical differences in cell proportions between sample groups were evaluated by Repeated Measures ANOVA using base R v. 4.1.3. Beta values were converted to M-values for statistical modeling.

A linear model was fitted using the limma v. 3.50.3 with time point as a fixed effect and patient ID as a blocking variable to account for repeated measures. Significant DMPs were determined based on a combination of thresholds: raw *p*-value < 0.01, deltaBeta > 0.05, and a positive B-statistic. DMRs were identified using the DMRcate v. 2.8.5 and bumphunter v. 1.36.0 packages and considered significant if they had five or more CpGs with a mean deltaBeta > 0.05 and DMRcate minimum smoothed false discovery rate (FDR) or Bumphunter permutation-based *p*-value < 0.05.

### 4.3. Transcriptome Analysis

PBMCs were lysed in the TRIzol reagent (Thermo Fisher Scientific). Total RNA was extracted from lysates using the PureLink RNA Micro Kit (Thermo Fisher Scientific). RNA quality was assessed with the RNA 6000 Pico Kit on a Bioanalyzer 2100 (Agilent Technologies, Santa Clara, CA, USA), resulting in a mean RIN of 9.4. Ribosomal RNA was depleted using the RiboCop rRNA Depletion Kit (Lexogen GmbH, Vienna, Austria). cDNA libraries were prepared with the NEBNext Directional Ultra II RNA-seq Kit (New England Biolabs, Ipswich, MA, USA). Library quality was evaluated with the High Sensitivity DNA Kit on a Bioanalyzer 2100 (Agilent Technologies), and concentrations were measured by qPCR using a CFX96 Touch Real-Time PCR Detection System (Bio-Rad Laboratories, Hercules, CA, USA). Sequencing was performed on a HiSeq platform (Illumina) with 2 × 100 bp paired-end reads.

Quality control of raw sequencing reads was assessed with FASTQC v. 0.12.0. Low-quality nucleotides were trimmed from the raw fastq files using Trimmomatic v. 0.39. Samples were mapped to the primary Genome assembly GRCh38 with the Gencode annotation using STAR v. 2.7.6a. Reads per gene were counted using the GeneCounts option of STAR software. DESeq2 v. 1.32.0 was used for their normalization.

### 4.4. Network and Gene Set Enrichment Analysis, Data Visualization

An interaction network of DMP-containing genes was constructed using the STRING 12.0 web service [32]. Gene set enrichment analysis of the LCC of the network was performed in pathways annotated in the KEGG database using the ShinyGO v.0.82 [33]. KEGG terms with FDR-corrected *p*-values less than 0.05 were considered significant.

Ggplot2 v. 3.5.2, as well as standard tools of the R programming environment v. 4.1.3, were used for data visualization.

## 5. Conclusions

This pilot study provides the first evidence that GA therapy induces dynamic, locus-specific DNA methylation changes in PBMCs from MS patients, with distinct temporal patterns suggesting a biphasic therapeutic response. The enrichment of DMPs in regulatory genomic regions, their integration into functionally interconnected networks, and significant correlations with gene expression collectively support the biological relevance of these epigenetic modifications. The identification of HLA-*DMA*, *PDE4A*, and *SMOX*, genes with established roles in MS-associated antigen presentation, immunoregulation, and neuroinflammation, opens new avenues for understanding GA’s multifaceted mechanisms of action. These findings, obtained with a limited budget, lay the groundwork for larger longitudinal, hypothesis-driven studies to validate GA-associated epigenetic signatures and explore their utility as biomarkers for monitoring therapeutic response and assessing bioequivalence of generic GA products.

## Figures and Tables

**Table 1:** Characteristics of MS patients included in the study.

Characteristic	Value
Number of patients, *n*	4
Female, *n* (%)	4 (100.0)
Mean age at MS onset, years ± SD	22.3 ± 3.8
Symptoms at onset (*n*)	Sensory symptoms (2), optic neuritis (1), neuropsychiatric symptoms (1)
Mean EDSS at the beginning of the study ± SD	2.0 ± 1.1

EDSS—expanded disability status scale; SD—standard deviation.

## Data Availability

Array data on DNA methylation in PBMCs are available in the Gene Expression Omnibus (GEO) database under accession number GSE310754; RNA-seq data are available from the corresponding author upon reasonable request.

## Supplementary Materials

The following supporting information can be downloaded at: https://www.mdpi.com/article/10.3390/ijms27104615/s1.

## Abbreviations

The following abbreviations are used in this manuscript:

DMP	Differentially methylated position
DMR	Differentially methylated region
DMT	Disease-modifying therapy
GA	Glatiramer acetate
LCC	Largest connected component
MS	Multiple sclerosis
PBMC	Peripheral blood mononuclear cells
