# Supplementary material for: Glatiramer Acetate Therapy Induces DNA Methylation Changes in Immune Cells of Multiple Sclerosis Patients: A Pilot Study
Source: Int J Mol Sci. 2026 May 21;27(10):4615. doi: 10.3390/ijms27104615 (PMC13207739; doi:10.3390/ijms27104615)
Supplement: Supplementary file 1 [file Supplementary_Figures.pdf › Supplementary_Figures.pdf]

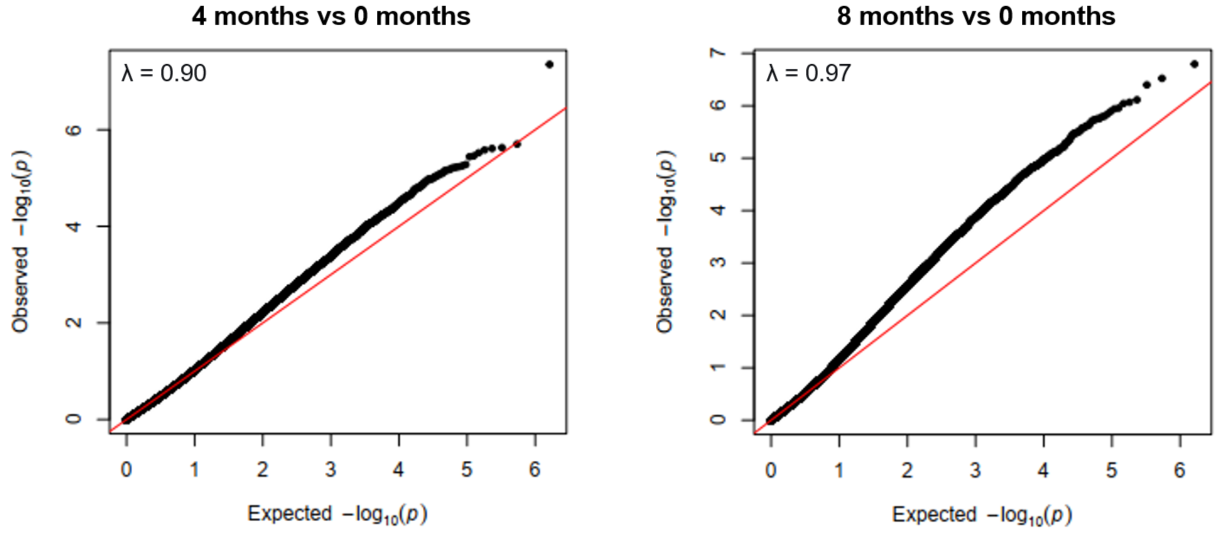

Figure S1. QQ-plots showing the observed *vs* expected –$\log_{10}$(p-values) for the analysis of differential DNA methylation. Reference diagonal lines are shown in red.

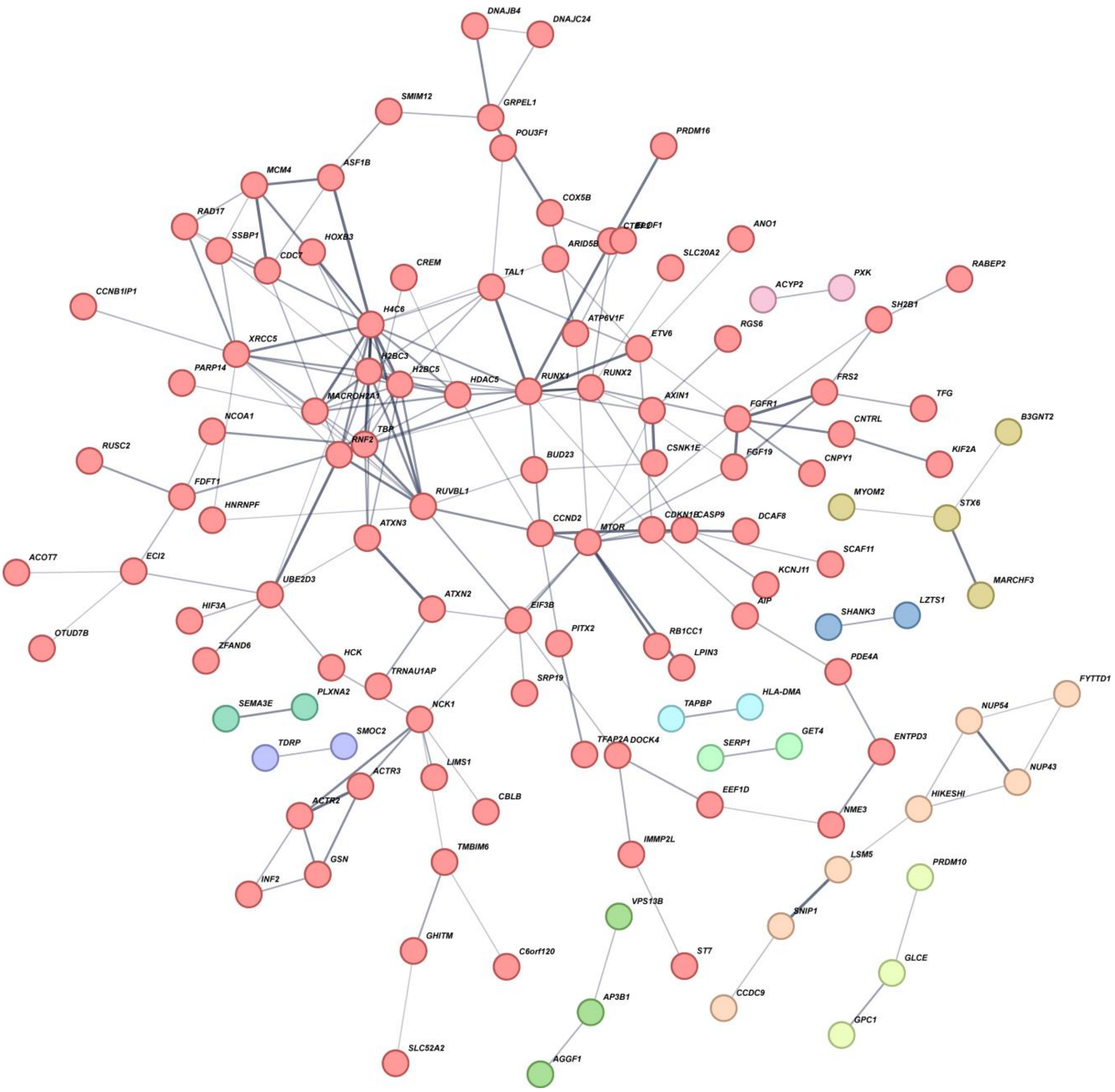

Figure S2. Eleven connected components of a STRING interaction network of all identified DMP-containing genes. Nodes of each connected component have the same color. Edge line thickness indicates the strength of data support.
